# Supplementary material for: Adaptation of retired older adult return migrants in their place of origin in the Yogyakarta Special Region, Indonesia
Source: PLoS One. 2025 Aug 6;20(8):e0328038. doi: 10.1371/journal.pone.0328038 (PMC12327630; doi:10.1371/journal.pone.0328038)
Supplement: Attachment — Submitted filename: Response to Reviewers 22 June 2025.docx [file pone.0328038.s003.docx]

**Response to Reviewers**

| Reviewers | Comments | Responses |
| --- | --- | --- |
| Reviewer#1 | Characterisation of the participants in this study needs to be framed more definitively as a study of well-educated and high-achiever retirees’ adaptation strategies on return to Yogyakarta (DIY) as their place of origin. The participants are over-represented in the Indonesian education achievement data. While the participants’ educational attainment varied from senior high to tertiary school graduates; the country’s education data shows (as of 2023) just around 40% of Indonesians aged 15 years and above had completed senior high school or more. | I framed the study by explaining that retired older adult return migrant are those who have attained higher level of education in following paragraph:  This study focuses on older adults who relocated to their place of origin immediately after retirement or during the subsequent period. In contrast to the general Indonesian population, the retired older adults in this study demonstrate a higher educational attainment. National data from 2024 indicate that only 41% of the population has attained secondary or higher education (55). The higher educational attainment among these retired older adults is likely due to the educational requirements of their former employment. Furthermore, the retired older adults in this study represent a relatively economically successful cohort, as their post-retirement financial capacity enables them to afford returning to their place of origin. (1^st^ paragraph of page 5) |
|  | It is written in the text DIY as “an ideal location” for retired older adult to reside. The question is about consequences of mal-adaptation associated with its custodianship of customary values. It would greatly benefit the text if you specified which social issues “potentially disrupting the harmony of a social environment” | This was my mistake in writing this sentence in the following text: “Conversely, those who encounter challenges in adaptation may face social issues potentially disrupting the harmony of a social environment”. (page 6 in the initial manuscript).  As a matter of fact, challenges the individuals face do not potentially disrupt the harmony of social environment but it could disrupt individuals integration to social environment. |

| No | Comments | Responses |
| --- | --- | --- |
|  |  | Therefore, I have revised the sentence as follow: “In contrast, individuals experiencing difficulties in adapting may face social challenges that significantly hinder their social integration, potentially driving further out-migration from their place of origin”. (last sentence in 1^st^ paragraph of page 6) |
|  | Suggestion to add a section on policy recommendations. | I have add section on policy recommendation. (page 27) |
|  | Minor grammar corrections are needed:  1) Studies on the adaptation of older adults who migrate internally, particularly to their place of origin, have not been conducted in Indonesia, despite this group of population contribute to the number of return migrants (p.5.)  2) The Yogyakarta Special Region Province is a central of Javanese culture, apart from Solo, a city in Central Java Province (p.5). | I have done grammar correction as follow:   1. Despite the significant contribution of older adults to internal return migration in Indonesia, research on their adaptation experiences remains limited. (1^st^ sentence in last paragraph of page 4). 2. The Yogyakarta Special Region, along with Solo in Central Java Province, which before independence was under the Surakarta Sultanate, serves as a primary centre of Javanese culture (57). (1^st^ sentence of page 6) |
| Reviewer#2 | Research question do not appear in the paper | I have appeared the research question in the paper as follow:  Addressing this gap, the central research question of this study examines the adaptive strategies employed by retired older adults to navigate the social environment after returning to their place of origin, with a particular emphasis on their social reintegration within their communities, especially at the neighbourhood level, following retirement (53). (page 5) |

| No | Comments | Responses |
| --- | --- | --- |
|  | Rearrange the explanation on pages 13-14 | I have rearranged the explanation on page 13-14. |
|  | Discussion the adaptation strategies according to gender, education level, previous occupation and so on. | Discussion on adaptation strategies among gender, education level, previous occupation is in the following paragraph:  The older adult participants represented diverse socio-demographic and economic backgrounds but the adaptation strategies were remarkably similar. This uniformity is primarily attributed to adherence to Javanese cultural values maintained despite having left the places of origin long ago. These values are deeply embedded in the social fabric of Yogyakarta and are embraced by residents regardless of their socio-demographic or economic status. Furthermore, individuals who are not ethnically Javanese but have previously lived in the Special Region of Yogyakarta tend to adopt these cultural norms. These shared norms facilitate social adaptation among retired older adult returnees. (page 27 🡪 paragraph before Policy Recommendation) |
|  | The main weakness of the paper is repetition, particularly in introduction and literature review. | I have omitted the repetition on “Introduction” and “Literature Review”. |
|  | The final manuscript should be edited by a professional language editor | Final manuscript has been edited by a professional language editor (Cambridge Proofreading). I attach the certificate of English editing. |
|  | References:  In-text citations - need to be corrected in one place on page 21. | I have corrected the reference: (82) (page 21). |

Following is certificate of English editing
